# Supplementary material for: StrVCTVRE: A supervised learning method to predict the pathogenicity of human genome structural variants
Source: Am J Hum Genet. 2022 Jan 14;109(2):195–209. doi: 10.1016/j.ajhg.2021.12.007 (PMC8874149; doi:10.1016/j.ajhg.2021.12.007)
Supplement: Document S1. Figures S1–S14 and Table S1 [file mmc1.pdf]

The American Journal of Human Genetics, Volume *109*

Supplemental information

StrVCTVRE: A supervised learning method to predict the pathogenicity of human genome structural variants

Andrew G. Sharo, Zhiqiang Hu, Shamil R. Sunyaev, and Steven E. Brenner

Supplemental Figures

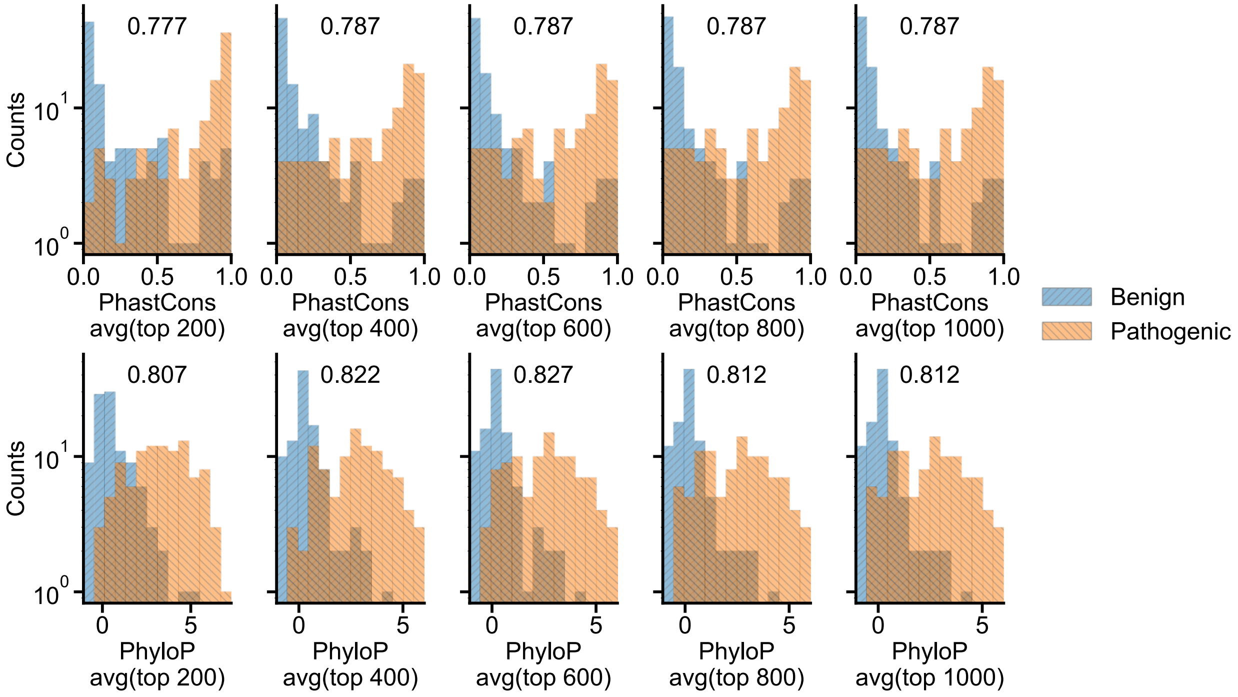

Fig. S1. Average of the top 400 PhyloP scores is one of the best indicators of SV pathogenicity. Comparison of PhyloP and PhastCons in differentiating between pathogenic and benign SVs. The columns represent different values of N, and the top row is PhastCons, while the bottom row is PhyloP. In each plot, the classification accuracy of a linear classifier is shown, and was calculated using a single feature: the average of the largest N PhyloP or PhastCons values among all positions overlapped by the SV. Each plot uses the same underlying SVs and shows a histogram of SV features.

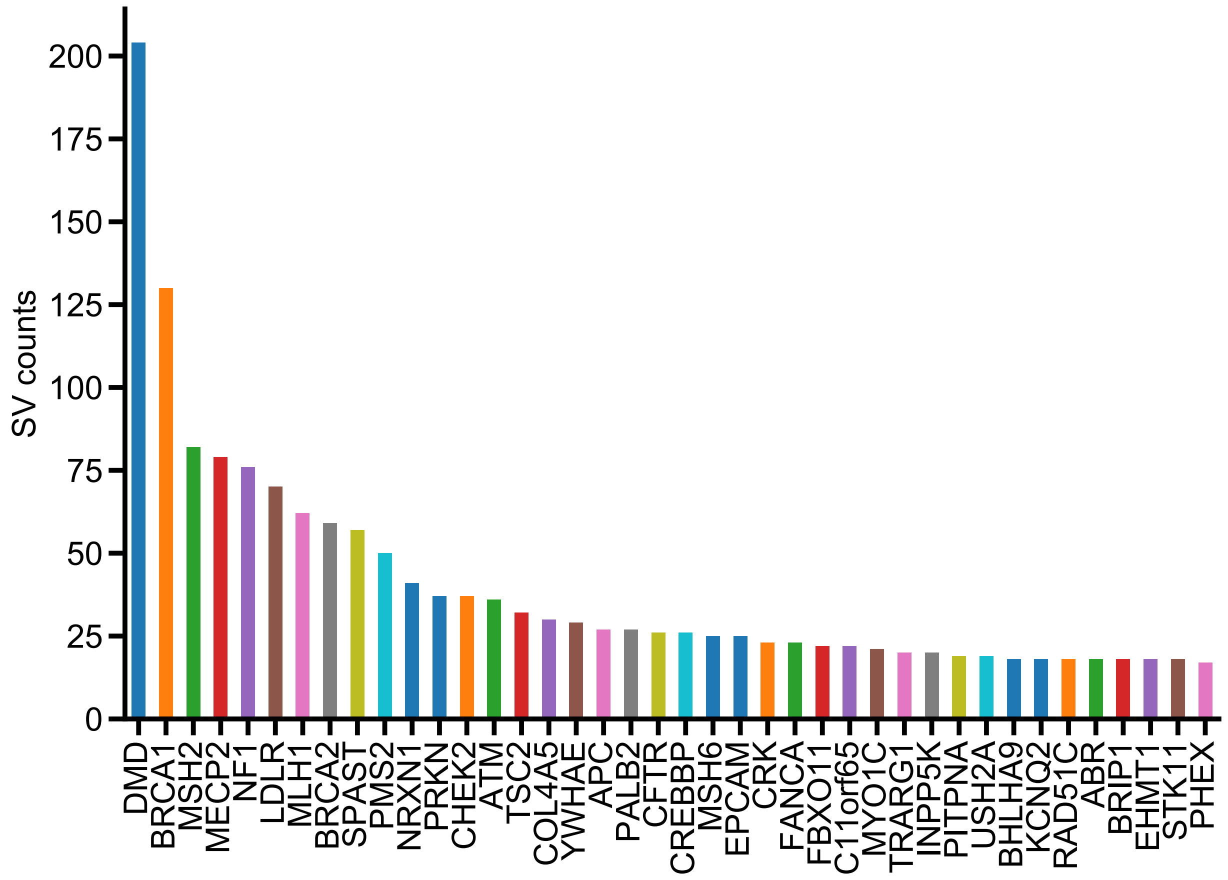

Fig. S2. Certain well-studied genes were overrepresented in our dataset. This plot shows the number of SVs overlapping each gene in the pathogenic ClinVar SVs (only showing the top 40 genes). For context, the median number of SVs overlapping each gene in our training dataset of pathogenic ClinVar SVs is 1.

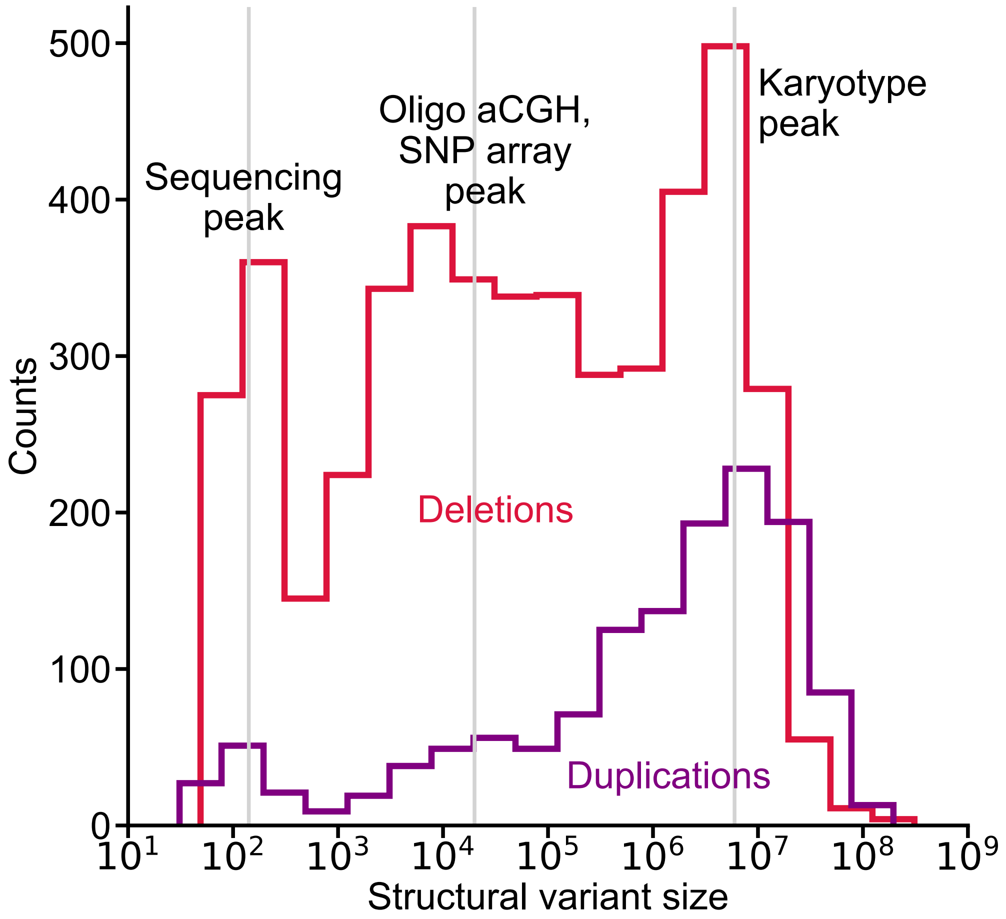

Fig. S3. Histogram of pathogenic SVs shows acquisition bias in SV size. Pathogenic SVs collected from ClinVar display three peaks consistent across both deletions and duplications. These peaks roughly correspond to the sensitivity range for three major methods to identify SVs: sequencing, oligo aCGH/SNP array, and karyotyping.

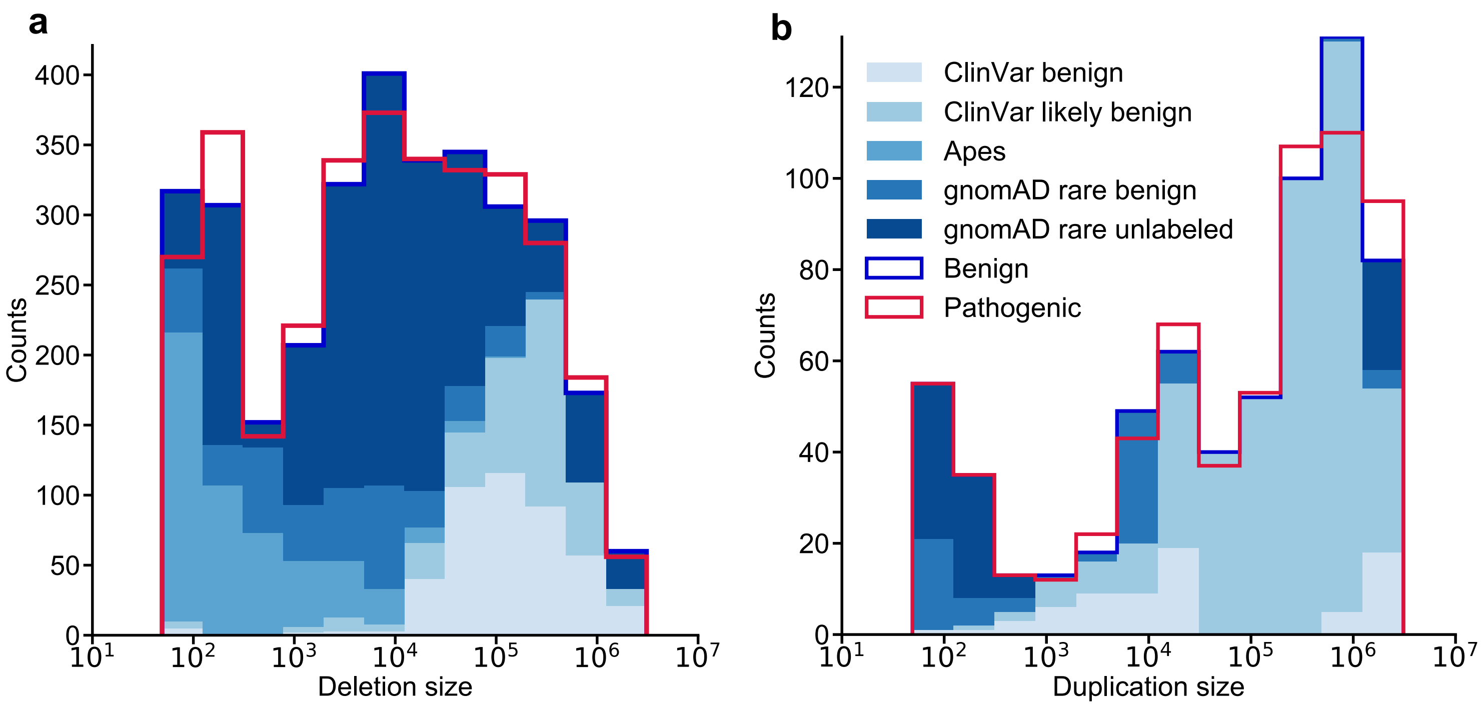

Fig. S4. Drawing SVs from multiple datasets allowed StrVCTVRE to train on a balanced dataset of SVs across a large size range. This is a more detailed version of Fig. 3. **a** Benign deletions were composed of 14% ClinVar benign, 12% ClinVar likely benign, 16% apes, 12% gnomAD rare benign, and 46% gnomAD rare unlabeled. **b** Benign duplications were composed of 11% ClinVar benign, 64% ClinVar likely benign, 11% gnomAD rare benign, and 14% gnomAD rare unlabeled.

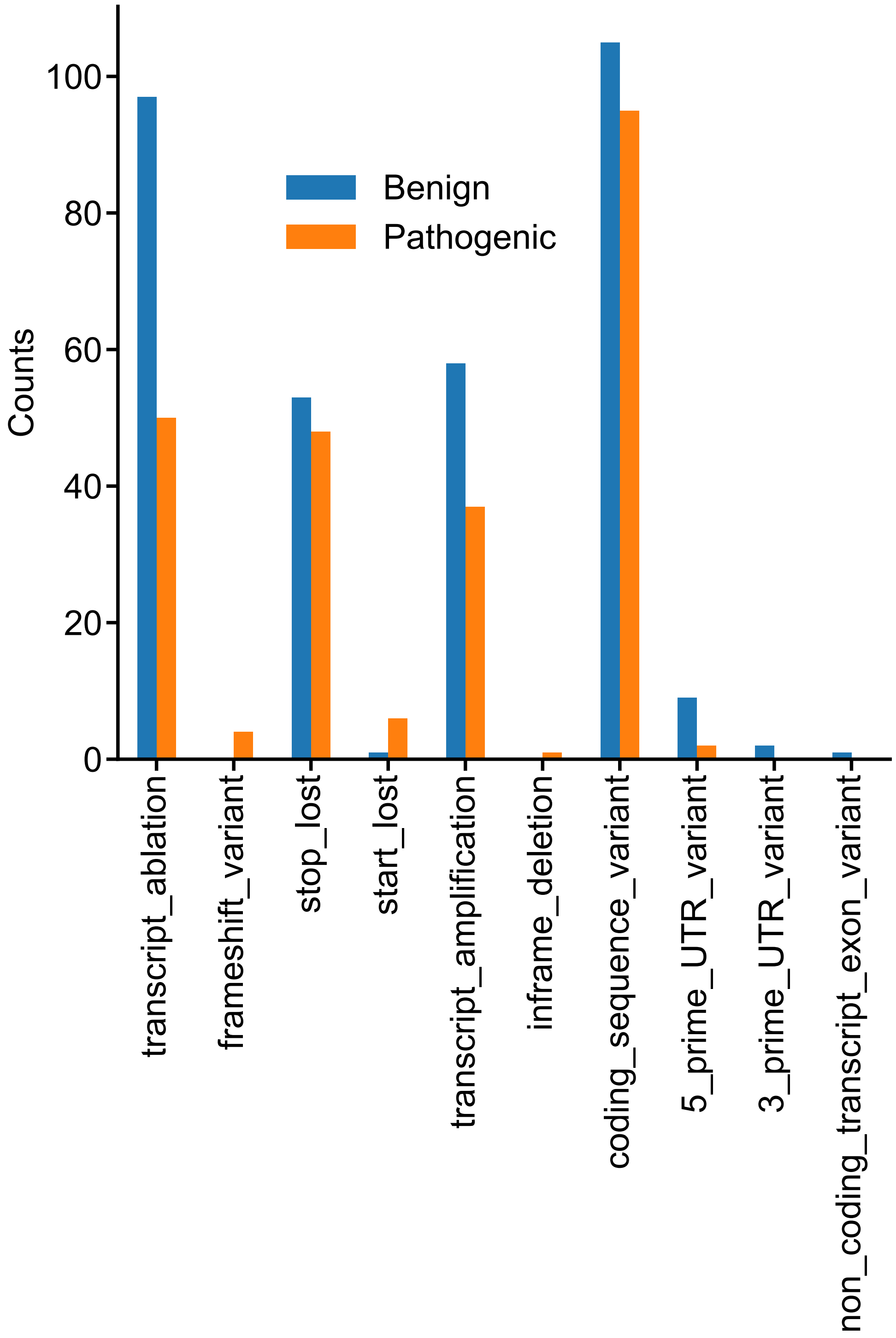

Fig. S5. Number of ClinVar test SVs (chromosomes 1, 3, 5, 7; from Fig. 2b) that VEP annotated with various sequence ontology terms. VEP classified more benign SVs than pathogenic SVs as transcript_ablation, leading to its poor performance in SV classification. Categories are ordered from left to right in descending deleteriousness.

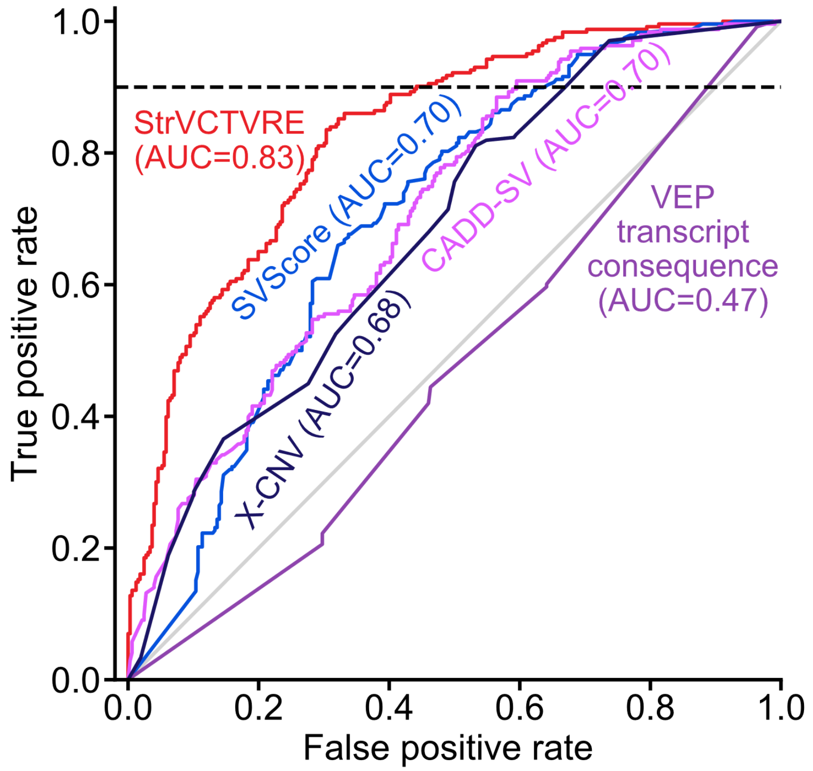

Fig. S6. Extended version of Fig. 2b. Receiver operating characteristic comparing StrVCTVRE (red) to SVScore (medium blue), CADD-SV (pink), X-CNV (dark blue), and VEP (purple) on a held-out test set comprised of ClinVar SVs on chromosomes 1, 3, 5, and 7.

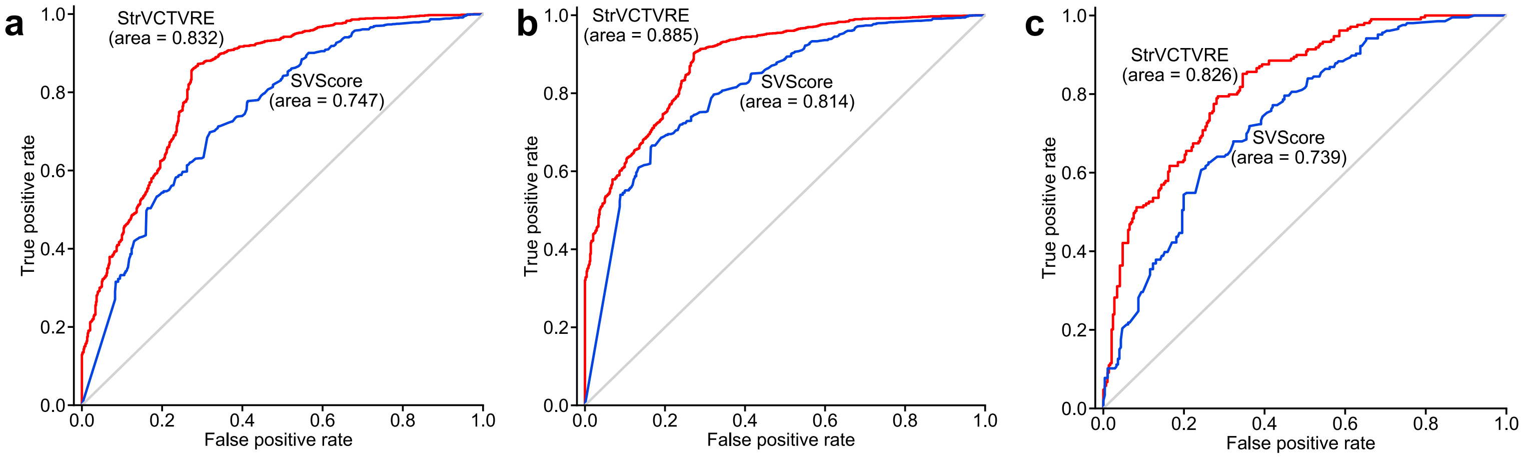

Fig. S7. StrVCTVRE and SVScore performance comparison on various held-out test datasets (ClinVar SVs on chromosomes 1, 3, 5, 7). **a** Duplicates and common variants were not removed from the test data. **b** Duplicates and common variants were not removed from the test data, and no size limit was imposed. **c** Duplicates and common variants were removed from the test data, and only SVs smaller than 1 Mb were included.

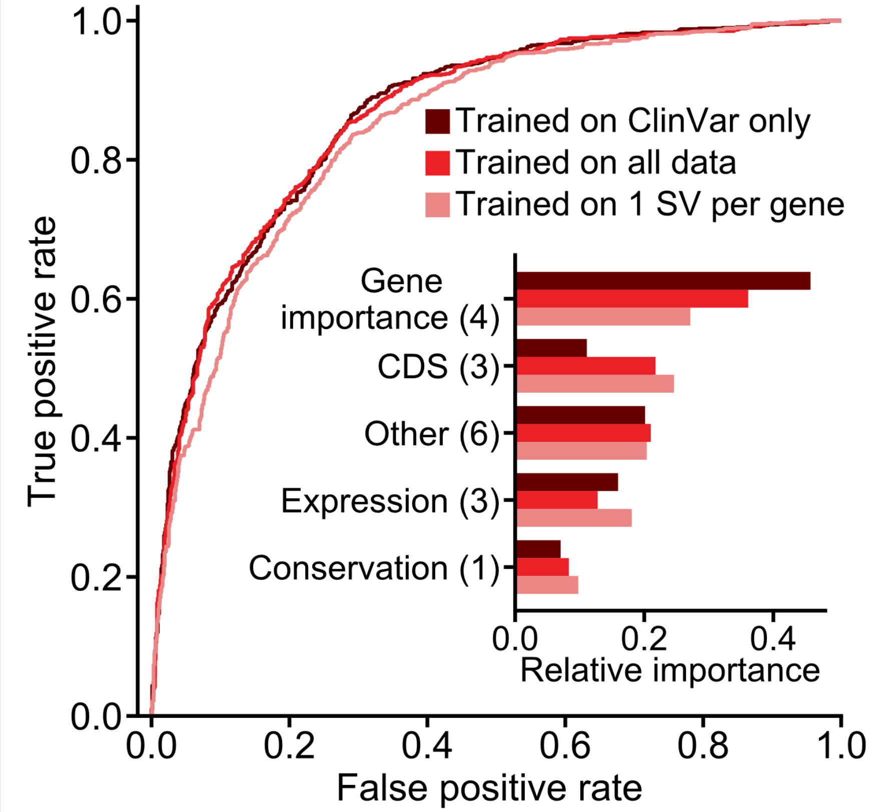

Fig. S8. Receiver operating characteristic comparing StrVCTVRE models trained on three different datasets: ClinVar in dark red, all data (ClinVar, SVs common to apes but not humans, and rare gnomAD SVs) in medium red, and a dataset in which each gene is overlapped by at most one SV in light red. The feature importances of the classifier trained on the one SV per gene dataset (light red) are more evently distributed than the other two datasets, but the AUC performance is decreased by 0.02 when this classifier is tested on held-out SVs.

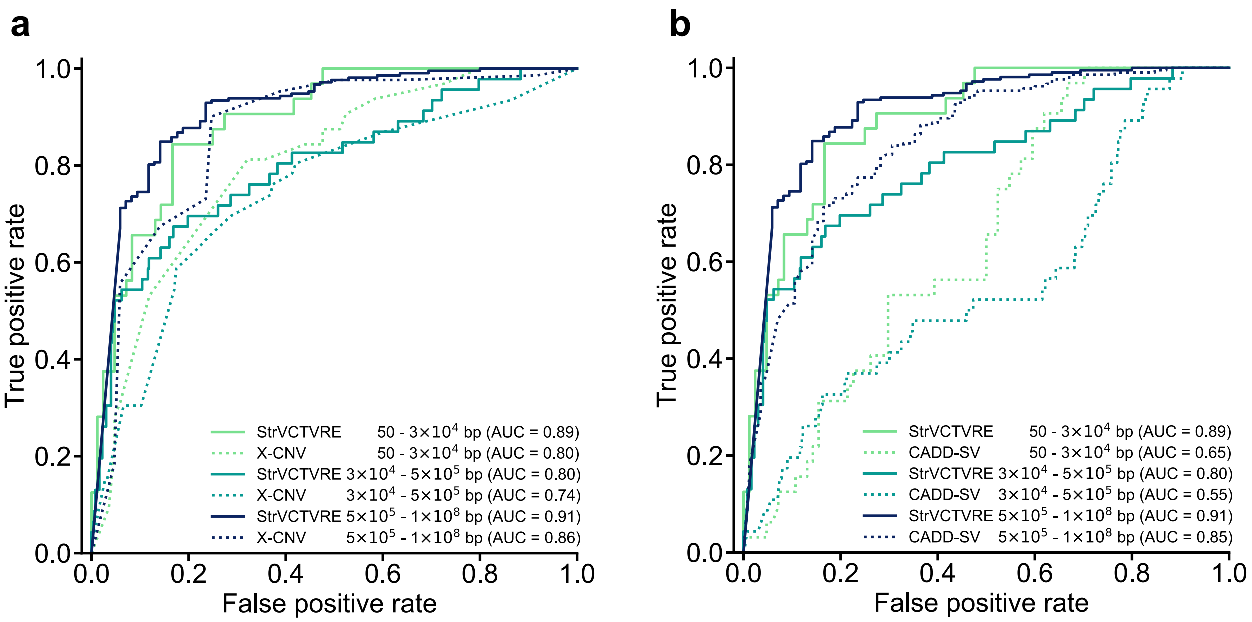

Fig. S9. Extended version of Fig. 4a. Comparison of StrVCTVRE with X-CNV and CADD-SV on a set of DECIPHER SVs across three size ranges. **a** StrVCTVRE (solid lines) performance compared to X-CNV (dotted lines). Line color denotes SV size range. **b** StrVCTVRE (solid lines) performance compared to CADD-SV (dotted lines). Line color denotes SV size range.

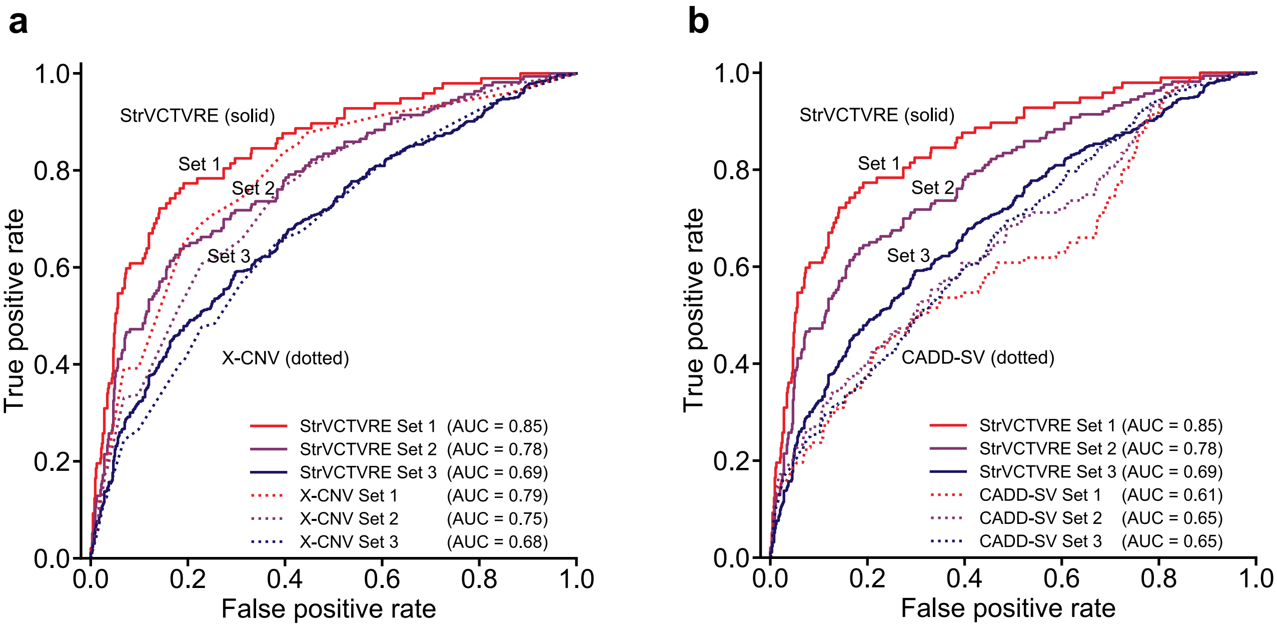

Fig. S10. Extended version of Fig. 4b. Comparison of StrVCTVRE with X-CNV and CADD-SV on a set of DECIPHER SVs with varying levels of contribution to proband phenotype. SV contribution to proband phenotype increases from set 3 (includes less confidently classified SVs) to set 2 and from set 2 to set 1 (most confidently classified SVs). **a** StrVCTVRE (solid lines) performance compared to X-CNV (dotted lines). **b** StrVCTVRE (solid lines) performance compared to CADD-SV (dotted lines).

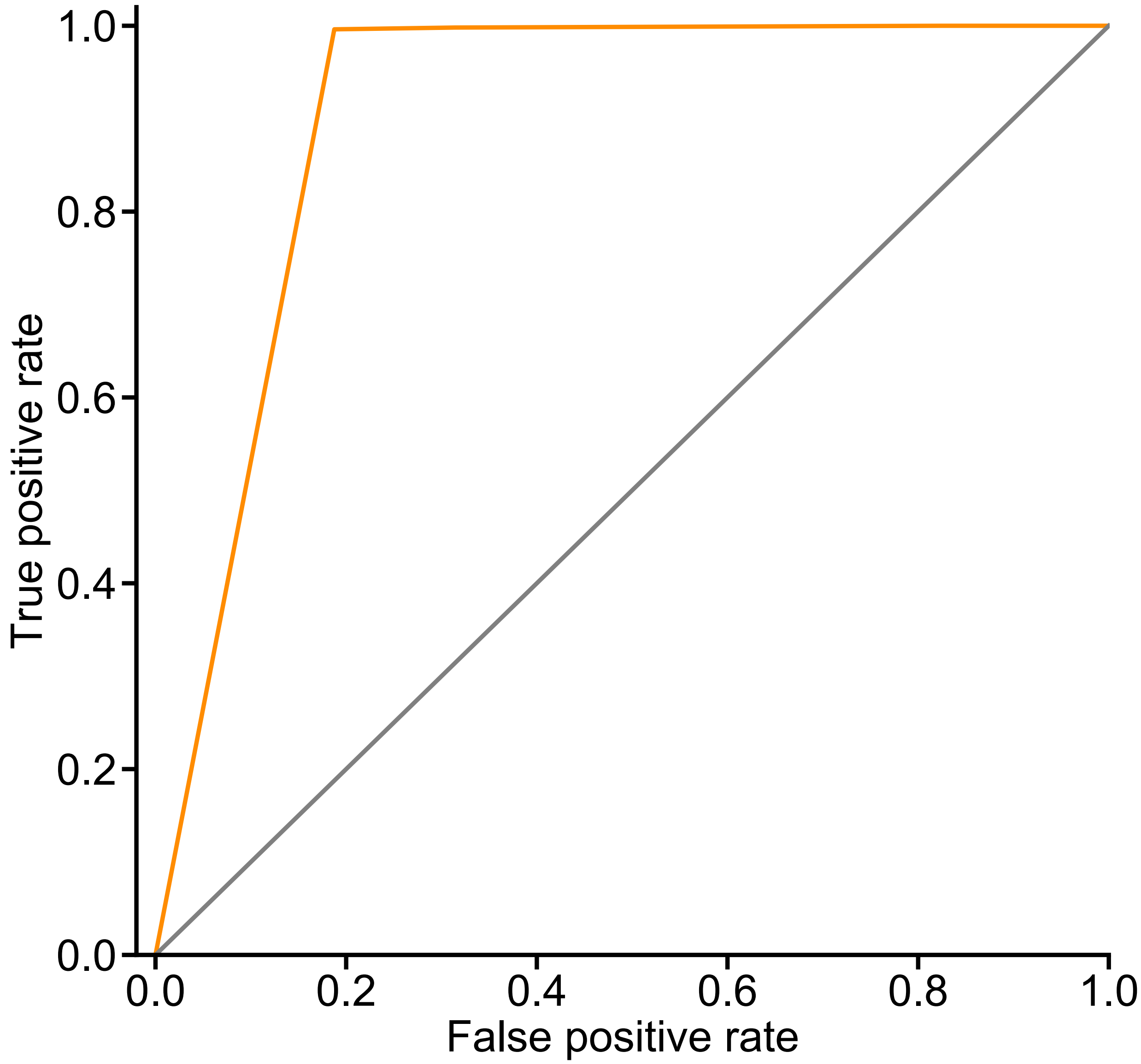

Fig. S11. AnnotSV excelled when tested on ClinVar variants that have a high degree of overlap with its cataloged variants. AnnotSV (orange) has an AUC of 0.905 when tested on 1042 ClinVar pathogenic variants and 867 ClinVar benign variants. It predicts nearly all pathogenic variants as pathogenic, along with 19% of the benign variants. The gray line represents chance.

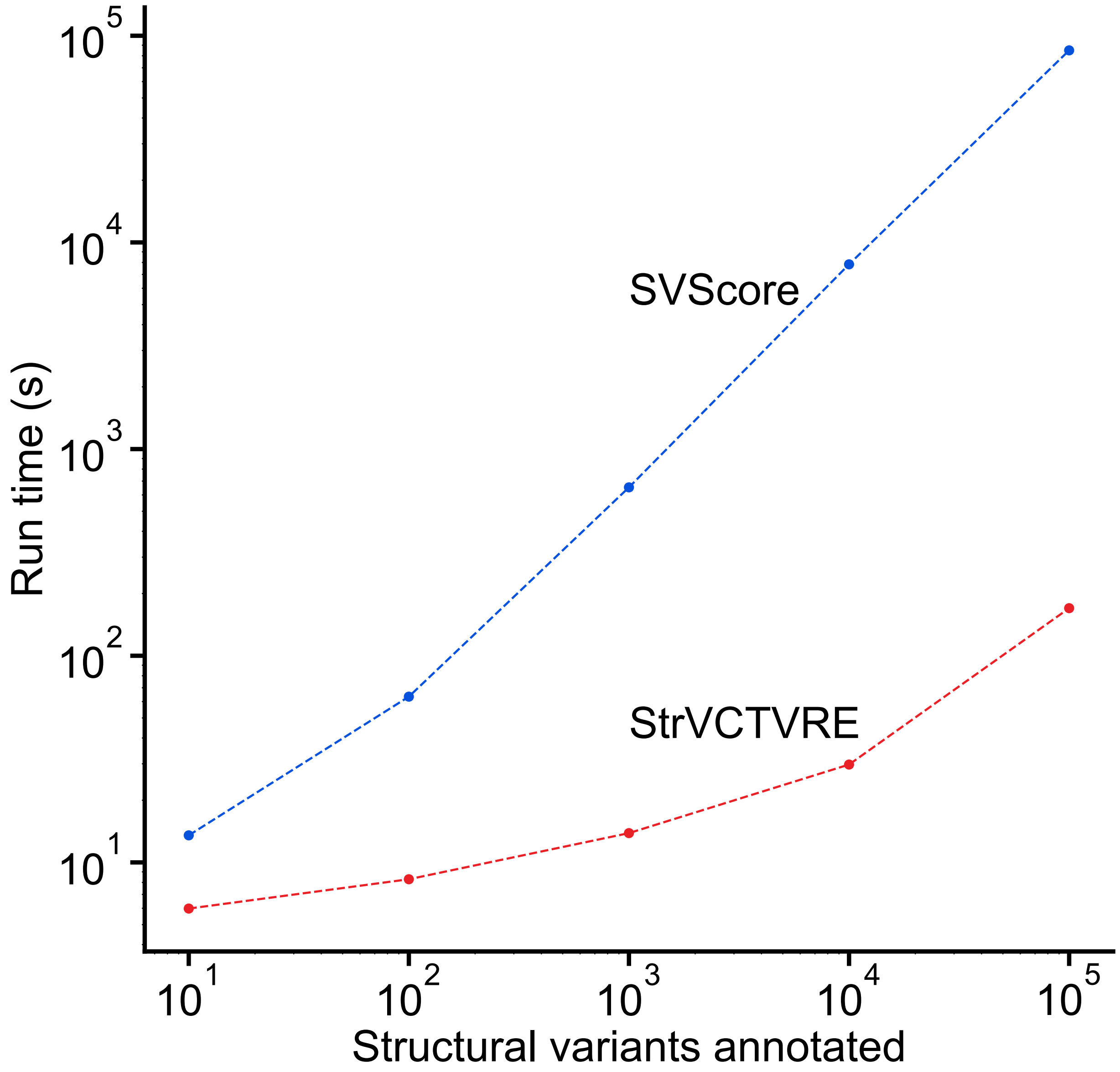

Fig. S12. Comparison of time required for StrVCTVRE and SVScore to annotate the same N structural variants. Each method was run on a 64-core Linux server with 2.6GHz Intel Xeon CPUs.

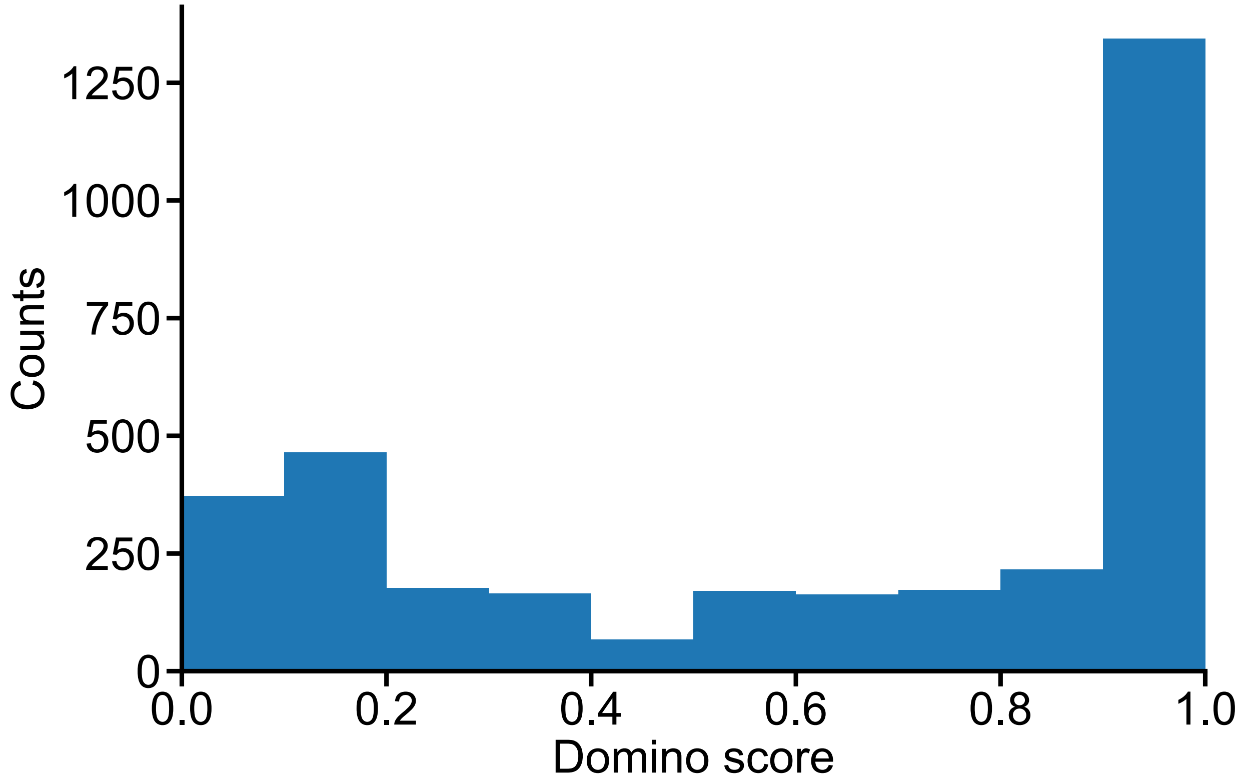

Fig. S13. Histogram of the predicted dominance of pathogenic SVs used in training. Predicted dominance was calculated for each SV by taking the maximum Domino(1) score of all genes the SV overlaps. SVs with larger Domino scores are more likely to have a dominant effect. Using a threshold of 0.5, 62% of pathogenic SVs are predicted to have a dominant effect. This analysis excludes SVs on sex chromosomes as they are not scored by Domino.

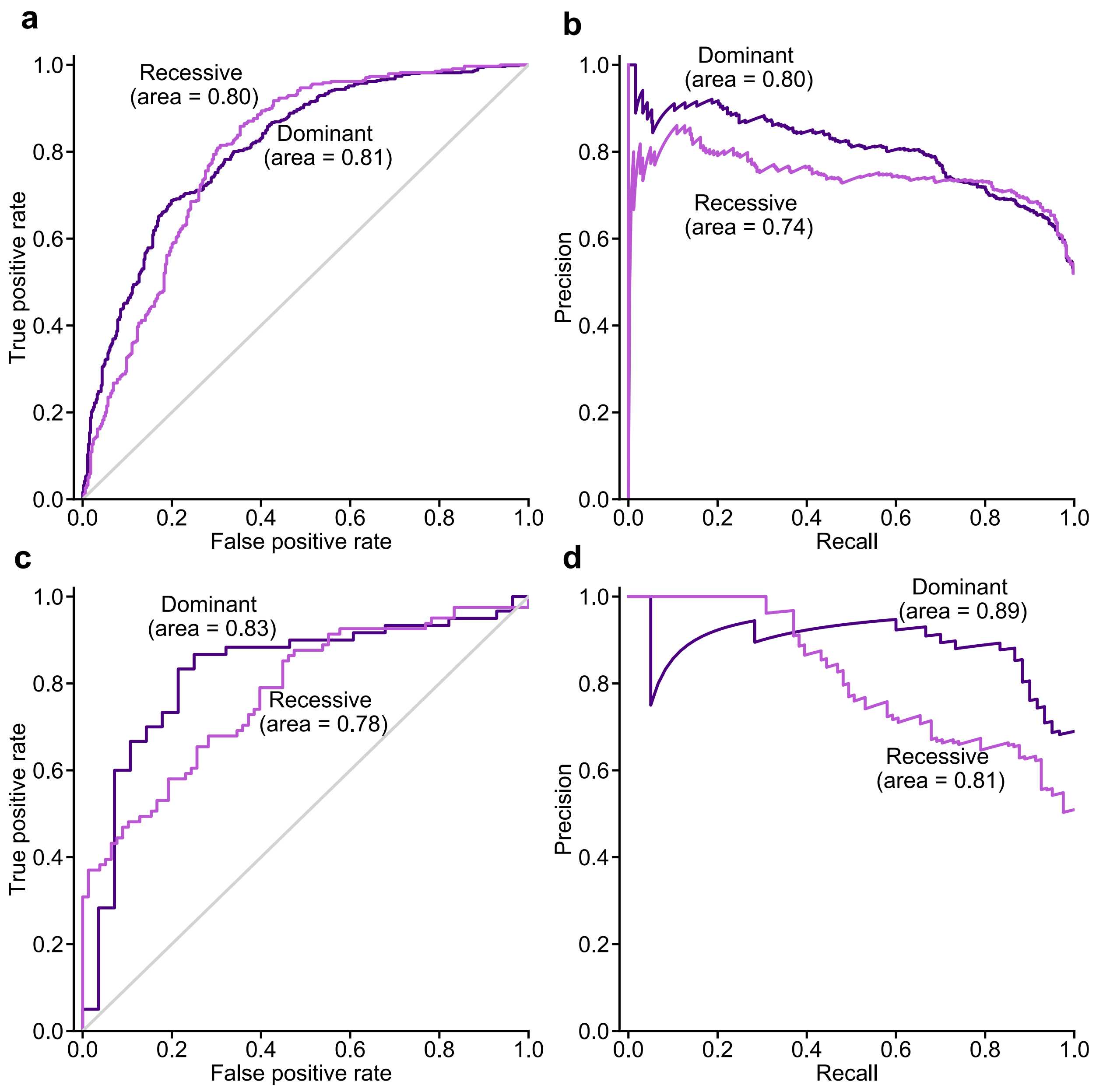

Fig. S14. ROC and Precision-Recall Curve (PRC) plots comparing StrVCTVRE's performance on recessive and dominant SVs. **a** ROC and **b** PRC plot of StrVCTVRE's performance on SVs in predicted dominant and recessive genes, using a Domino(1) threshold of 0.5 to separate dominant from recessive genes. **c** ROC and **d** PRC plot of StrVCTVRE's performance on SVs overlapping high-confidence recessive or dominant genes identified by Balick et al(2). Overall, StrVCTVRE performs similarly on SVs in both dominant and recessive genes. There may be differences in performance at low sensitivity/recall but they are not consistent across datasets.

Supplemental Tables

chrom	start	end	svtype	StrVCTVRE Score
chr1	145687715	146020436	DEL	0.768
chr1	202434559	202604719	DEL	0.673
chr2	111994154	111994818	DEL	0.569
chr2	219420423	219426832	DEL	0.511
chr6	10791578	10791945	DEL	0.276
chr6	64997485	65057825	DEL	0.634
chr6	64997485	65057825	DEL	0.634
chr10	27043329	27067384	DUP	0.699
chr13	23320540	23320858	DEL	0.3
chr15	42321688	42360212	DEL	0.502
chr15	42384386	42394439	DEL	0.619
chr16	28486250	28486550	DEL	0.571
chr17	7454200	7454618	DEL	0.567
chr17	50170063	50170449	DEL	0.471
chr19	54105500	54126715	DEL	0.678
chr19	54114345	54129468	DEL	0.753
chr19	54121739	54131817	DEL	0.847
chrX	31627576	31679684	DEL	0.835
chrX	31819878	31968612	DEL	0.847
chrX	32545062	32699391	DEL	0.823
chrX	154379176	154381621	DEL	0.601

Table S1. Subset of the 34 CMG clinical SVs used to evaluate the StrVCTVRE 90% sensitivity threshold. Due to data restrictions, the full list of SVs is not available. See ‘Data and code availability’ for more details.

Supplemental References

1. Quinodoz M, Royer-Bertrand B, Cisarova K, Di Gioia SA, Superti-Furga A, Rivolta C. DOMINO: using machine learning to predict genes associated with dominant disorders. The American Journal of Human Genetics. 2017;101(4):623-9.
2. Balick DJ, Jordan DM, Sunyaev S, Do R. Overcoming constraints on the detection of recessive selection in human genes from population frequency data. bioRxiv. 2021.
